# Psychological resources and interventions for teachers’ emotional competence and well-being: a systematic review

**DOI:** 10.3389/fpsyg.2025.1640968

**Published:** 2025-08-15

**Authors:** Brenda Cervellione, Ester Maria Concetta Lombardo, Calogero Iacolino

**Affiliations:** Department of Human and Social Sciences, Kore University of Enna, Enna, Italy

**Keywords:** emotional intelligence, reflective functioning, burnout, teachers’ well-being, psychological resources, systematic review, educational contexts

## Abstract

**Introduction:**

Teachers are increasingly exposed to emotional and organizational stressors that affect their psychological well-being and professional functioning. Emotional Intelligence (EI) and Reflective Functioning (RF) have emerged as key protective factors in preventing burnout and promoting Quality of Life (QoL) in educational contexts.

**Methods:**

This systematic review followed the PRISMA 2020 guidelines and synthesized findings from 42 empirical studies published between 2010 and 2024. Studies were identified through comprehensive searches of EBSCOhost, PubMed, and Google Scholar. Both psychological interventions and observational studies exploring the relationship between EI, RF, and teacher well-being were included.

**Results:**

Intervention studies showed that programs targeting emotional regulation, mindfulness, and reflective capacity significantly improved teachers’ emotional competencies and psychological resilience. Observational studies further supported the role of EI and RF in reducing burnout and enhancing QoL, while also highlighting the influence of personality traits and contextual moderators. However, methodological heterogeneity, limited use of longitudinal designs, and variation in outcome measures constrained generalizability.

**Discussion:**

Findings underscore the importance of promoting psychological resources and structured interventions to support teachers’ emotional well-being and professional sustainability. Future research should prioritize standardized, longitudinal, and culturally sensitive approaches to better inform evidence-based practice in educational settings.

## Introduction

The teaching profession is widely recognized as both emotionally and cognitively demanding, frequently exposing educators to chronic stress and emotional exhaustion. Teachers must navigate complex interpersonal dynamics, meet increasing academic standards, and manage escalating bureaucratic requirements, all of which can significantly affect their psychological well-being (Agyapong et al., 2023; Jose and Kushwaha, 2024; Iacolino et al., 2023). These stressors are consistently associated with elevated levels of burnout (Belinda and Wei, 2024; Nizielski et al., 2013), decreased job satisfaction (Castro Silva et al., 2024), and diminished quality of life, rendering the profession particularly vulnerable to mental health challenges (Solih et al., 2024, Thomas, 2024, Zdanevych et al., 2024; Pandey and Sharma, 2023, Recktenvald and Donelli, 2019).

In response, growing scholarly attention has focused on identifying psychological resources that may buffer the adverse effects of these occupational stressors. Among the most studied constructs are Emotional Intelligence (EI) and Reflective Functioning (RF). EI refers to the ability to perceive, regulate, and utilize emotional information effectively (Salovey and Mayer, 1990), and is considered a key factor in managing classroom dynamics and interpersonal relationships (Lucas-Mangas et al., 2022; Zewude et al., 2024). It is positively associated with psychological capital, stress resilience, and professional well-being in educational settings (Iacolino et al., 2023; Sánchez-Álvarez et al., 2016). RF, closely related to the concept of mentalization, concerns the ability to understand one’s own and others’ behavior in terms of underlying mental states (Fonagy and Target, 1997). Teachers with stronger RF tend to experience greater emotional balance, interpersonal attunement, and professional satisfaction, while impairments in RF—such as hypermentalizing or hypomentalizing—have been associated with emotional dysregulation and burnout (Safiye et al., 2023; Schwarzer et al., 2025).

Psychological interventions targeting EI and RF, including mindfulness-based stress reduction (MBSR), cognitive-behavioral therapy (CBT), and emotional regulation training, have shown promising results in reducing stress and burnout among teachers (Agyapong et al., 2023). Notably, both pre-service and in-service teachers may benefit from such interventions, although the effectiveness of these programs may vary depending on contextual and individual factors. Improvements in EI, in particular, have been associated not only with greater psychological well-being but also with enhanced organizational commitment and job performance (Li et al., 2024). These interventions are typically designed to promote emotional regulation, self-awareness, and relational competence, and are applicable in both pre-service and in-service teacher training (Messina-Albarenque et al., 2024).

Despite a growing body of empirical work on these topics, few reviews have systematically examined the combined role of EI and RF—both as psychological constructs and as targets of intervention—in relation to teacher well-being.

In this review, we adopt psychological well-being as the central construct, defined according to Ryff’s multidimensional model encompassing autonomy, environmental mastery, personal growth, positive relations, purpose in life, and self-acceptance (Ryff, 1989). While the terms “emotional well-being” and “quality of life” are also found in the literature, we treat them as related but non-equivalent constructs. Quality of life, for instance, is conceptualized by the World Health Organization as an individual’s perception of their position in life within the context of their culture and value system, and is considered here as one dimension of broader psychological well-being (WHOQOL Group, 1995).

Existing reviews tend to focus on stress and burnout in isolation, often overlooking how these outcomes intersect with personality traits, reflective functioning, and broader constructs such as emotional regulation. Moreover, while many studies evaluate structured psychological interventions, others use observational or correlational designs to explore individual traits and coping styles in relation to well-being. These heterogeneous approaches reflect the complexity of teacher experience but have not been integrated in a unified synthesis.

This systematic review aims to address these gaps by:

(1) synthesizing empirical findings on both psychological interventions and psychological correlates associated with EI and RF;
(2) assessing their relationship with burnout and psychological well-being among teachers; and
(3) exploring how individual characteristics and contextual moderators may influence these outcomes.

By incorporating evidence from diverse research designs and conceptual frameworks, this review seeks to inform targeted, evidence-based practices to support teacher well-being and professional sustainability.

## Methods

### Inclusion and exclusion criteria

Studies were included in this review if they met the following criteria: (1) the population consisted of active or trainee teachers; (2) the study involved either psychological interventions aimed at improving Emotional Intelligence (EI) or Reflective Functioning (RF), or assessed these constructs through observational or correlational designs; (3) outcomes included EI, RF, burnout, personality traits, or quality of life, measured with empirically validated instruments; (4) the article was published in English between 2010 and 2024 to reflect current educational trends; (5) the study employed experimental, quasi-experimental, cross-sectional, or correlational research designs.

Studies with both interventional and non-interventional designs (e.g., correlational, cross-sectional, observational) were included to comprehensively capture the state of the literature on psychological factors influencing teachers’ well-being and to reflect the heterogeneity in available empirical evidence.

Exclusion criteria were: (1) opinion pieces, editorials, gray literature, and narrative reviews without empirical data; (2) studies involving populations other than teachers; (3) studies that did not focus on EI or RF or failed to measure relevant psychological outcomes; (4) articles with no full-text access.

### Information sources and search strategy

The search was conducted in the following academic databases: EBSCO (including Psychology and Behavioral Sciences Collection, APA PsycArticles, Education Research Complete, MEDLINE Complete, and CINAHL Complete), PubMed, and Google Scholar. Access to these sources was provided through the library system of the University of Enna “Kore.”

Search terms included combinations of the following keywords: “Emotional Intelligence,” “Reflective Functioning,” “Burnout,” “Personality Traits,” “Quality of Life,” “Teacher Psychological Training,” and “Teacher.” The search was restricted to publications from 2010 to 2024. Duplicate records were removed using EndNote bibliographic software prior to screening.

The screening was conducted in two phases. First, titles and abstracts were independently screened by two reviewers (BC, CI). Second, full texts were examined for eligibility. Discrepancies at both stages were resolved through discussion or, when necessary, with the involvement of a third reviewer (EMCL).

This review was conducted according to PRISMA 2020 guidelines (Page et al., 2021), and was not registered in PROSPERO.

A detailed summary of study characteristics, interventions, and outcome measures is provided in the Supplementary Tables 1–5.

A narrative quality appraisal was performed for all included studies, focusing on key methodological dimensions such as study design, psychometric validity of the instruments used, presence of control/comparison groups, and clarity of reported outcomes. Due to the heterogeneity of designs and aims, a formal risk of bias assessment tool (e.g., Cochrane, ROBINS-I) was not applied. Nevertheless, each study’s methodological strengths and limitations were documented thematically to support transparency and interpretative clarity (see Supplementary Table 5 for a synthesis).

### Study synthesis

Given the methodological heterogeneity of the included studies, a narrative synthesis approach was adopted (narrative-informed review), which is suitable for comparing findings across diverse research designs and theoretical orientations (Popay et al., 2006). The synthesis followed three iterative steps. First, studies were organized into thematic categories based on intervention type (e.g., mindfulness-based training, emotional skills workshops), research design (pre-post, cross-sectional), and primary outcomes. Second, comparisons were made within each category to highlight methodological differences and common findings. Third, cross-category analyses were conducted to identify relationships between intervention outcomes and moderating variables such as personality traits, school context, and cultural setting.

This review was not registered in PROSPERO, as preregistration is not currently required for narrative or exploratory reviews. No formal risk of bias assessment was conducted, in line with the conceptual aims of the synthesis, which focused on mapping key psychological constructs rather than evaluating intervention efficacy.

While no preregistration was conducted (given the narrative aims of the review), transparency and replicability were ensured through detailed reporting of methods, inclusion criteria, and Supplementary material.

**Figure 1:**
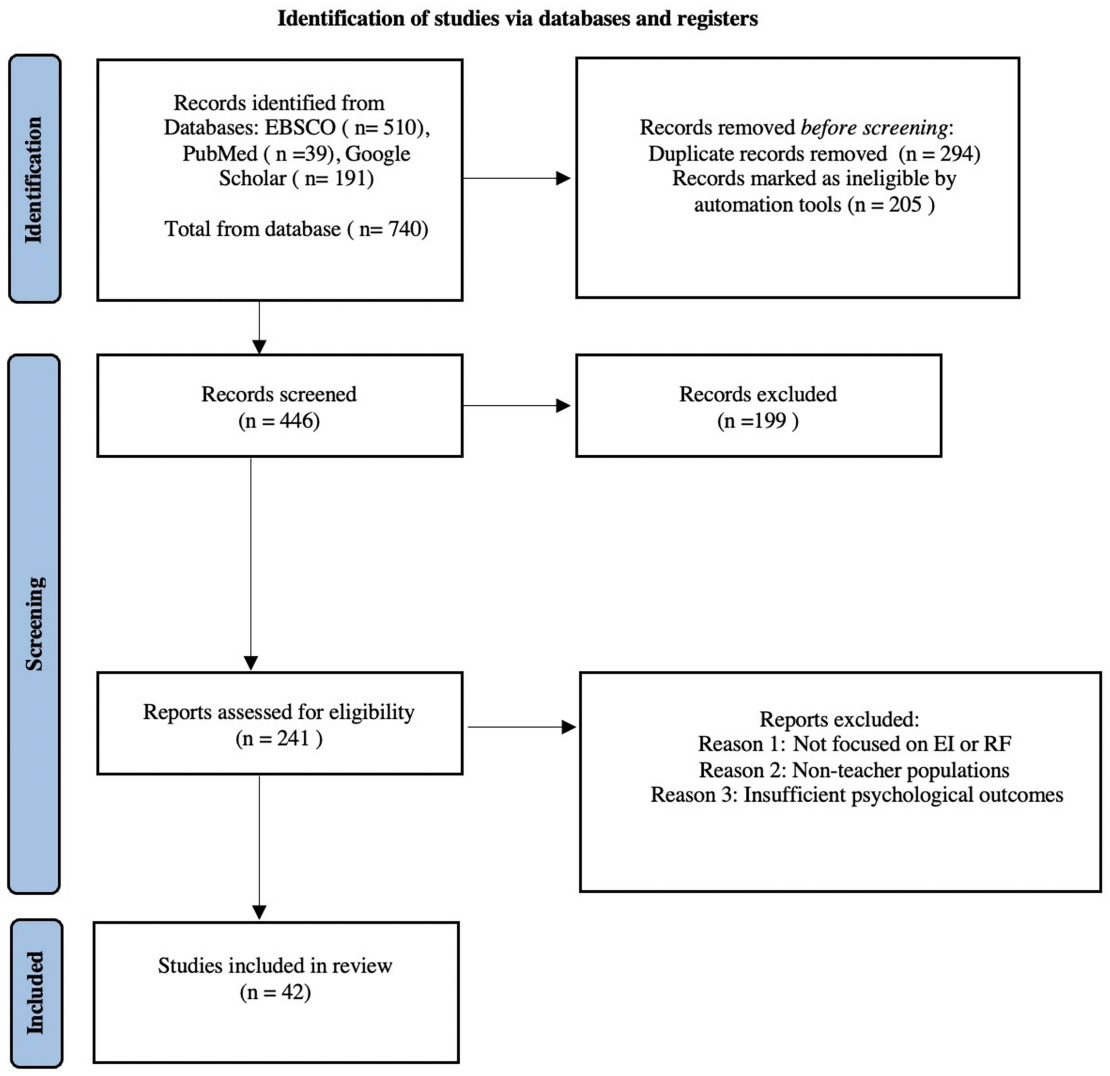
PRISMA 2020 flow diagram illustrating the selection process of studies included in the systematic review. The diagram shows the number of records identified, screened, excluded, and finally included, along with the main reasons for exclusion during the full-text assessment phase.

The PRISMA flow diagram (Figure 1) illustrates the selection process.

Excluded full-text articles (*n* = 199) were mainly removed due to lack of focus on EI/RF, non-teacher populations, or insufficient reporting of psychological outcomes.

Additionally, backward citation tracking was used to identify relevant studies cited in key reviews. No grey literature or unpublished theses were included.

### Characteristics of included studies

The studies included in this review exhibit substantial diversity in terms of research design, target population, and type of psychological intervention. A summary of key methodological characteristics is presented below.

Study Designs. The included studies employed a range of designs, including experimental, quasi-experimental, cross-sectional, and correlational approaches. Several studies used pre-post designs with control groups to assess the effectiveness of psychological interventions, while others investigated associations among emotional intelligence, reflective functioning, burnout, and quality of life through observational or correlational methods.

Sample. The samples consisted of teachers from various countries and educational levels, including primary and secondary school educators, pedagogy students, and pre-service teachers. Sample sizes ranged widely—from small groups of approximately 30 participants to larger cohorts exceeding 1,000. Most studies reported diverse demographics in terms of age and gender, although a few focused on specific subgroups.

Interventions. The psychological interventions analyzed included mindfulness and meditation programs, socio-psychological training, and emotional intelligence development courses. These interventions aimed to enhance teachers’ emotional regulation, relational skills, and psychological resilience. Some studies implemented interactive workshops and video-based training, while others focused on the assessment and development of reflective or socio-emotional competencies.

Control Groups. Where applicable, experimental studies incorporated control groups for comparative purposes. In some cases, control groups received no intervention; in others, they participated in alternative training programs or continued with standard curricular activities.

Measurement Timing. For studies with pre-post designs, outcomes were typically assessed at the conclusion of the intervention and, in some cases, again at follow-up points (e.g., 6 months later) to evaluate the durability of effects. Measures included both self-report instruments and structured observational tools, depending on the variables assessed.

A detailed overview of each study is available in the Supplementary Tables 1–4, which includes comprehensive data on study design, sample characteristics, intervention type, outcome measures, and main findings.

A narrative quality appraisal was also performed and is presented in the Supplementary Table 5, in accordance with the methodological recommendations of recent systematic reviews.

### General overview

The synthesis of findings reveals that psychological interventions targeting mindfulness, emotional intelligence (EI), and emotional regulation strategies consistently yielded positive outcomes across several dimensions of teacher well-being. These include reductions in burnout symptoms, increased job satisfaction, and improvements in overall Quality of Life (QoL). Quantitative studies employing pre-post designs reported average reductions in burnout ranging from 20 to 30%, while approximately 70% of the included studies showed statistically significant gains in psychological well-being indicators.

However, substantial variability emerged across studies in terms of methodological approach, cultural context, and population characteristics. For example, experimental studies tended to report more robust post-intervention effects, while correlational or cross-sectional designs primarily identified associations between EI or reflective functioning (RF) and outcomes such as emotional exhaustion or personal accomplishment, without establishing causality. Moreover, inconsistencies in how key constructs—particularly “well-being” and “quality of life”—were defined and measured complicated direct comparisons across studies.

These discrepancies underscore the importance of clarifying construct definitions and adopting more methodologically rigorous designs in future research. Tailoring interventions to cultural and contextual specificities, while improving standardization in outcome measurement, may enhance both the applicability and the scientific value of this emerging evidence base.

### Effects of psychological interventions on teachers’ emotional intelligence

Emotional intelligence (EI) plays a crucial role in managing the daily emotional demands of teaching, contributing to relational effectiveness and emotional resilience in school environments. Several studies in this review underscore the effectiveness of EI-focused interventions, with some incorporating follow-up assessments to evaluate long-term impact.

Kuk et al. (2015), for instance, implemented a video-based interpersonal training program with physical education students in Poland. Their results demonstrated sustained improvements in EI and social competence, confirmed at six-month follow-up. Although methodologically robust, the study’s generalizability is limited by its focus on a specific subset of university students, raising concerns about applicability across broader teaching contexts.

A complementary approach was adopted by Sankhayeva et al. (2021), who implemented socio-psychological training for pedagogy students in Kazakhstan. The program produced significant gains in EI and relational abilities. However, the lack of long-term follow-up limits understanding of the intervention’s durability, although the broader range of socio-emotional competencies assessed represents a methodological strength.

Cheng et al. (2022) expanded the scope by combining EI development with burnout and depression reduction through mindfulness training. Despite its modest sample size (70 kindergarten teachers), the intervention produced substantial improvements in both EI and psychological well-being. Unlike Kuk et al. and Sankhayeva et al., this study embraced a more holistic, integrated model—reinforcing the value of multi-target interventions in high-stress educational contexts.

Correlational studies also contributed meaningful insights. Arteaga-Cedeño et al. (2022), analyzing data from 351 teachers, identified age and work experience as significant predictors of emotional competence. While causality cannot be inferred, the findings underscore the importance of personal and demographic variables in tailoring EI programs.

Similarly, Mérida-López et al. (2023), with a large sample of 1,166 Spanish teachers, demonstrated that perceived stress mediates the relationship between EI and work engagement. The study’s mediation model supports the use of multifactorial interventions that combine EI enhancement with stress reduction strategies. However, as with many cross-sectional designs, causal conclusions remain limited.

Wu (2004) offered additional perspective by analyzing gender- and age-related differences in EI among Taiwanese teachers. Although no intervention was tested, the study identified important demographic patterns, reinforcing the need for demographic-specific adaptation in EI programming.

Together, these findings suggest that while targeted EI interventions are promising, their impact may be maximized when adapted to individual and contextual factors—such as demographic characteristics, occupational roles, and cultural settings. Longitudinal studies and randomized controlled trials are needed to validate and expand upon these promising trends.

### The impact of psychological interventions on teachers’ reflective function

Reflective functioning (RF)—conceptualized as the capacity to interpret one’s own and others’ behaviors by attributing underlying mental states—has emerged as a key modifiable psychological function that contributes to burnout reduction and improved relational efficacy in teaching environments. Despite receiving comparatively less attention than emotional intelligence, RF offers valuable insights into teacher well-being, particularly in terms of emotional regulation and interpersonal dynamics.

Dexter and Wall (2021) examined the mediating role of self-efficacy between RF and burnout in a cross-sectional design. While their results suggested that stronger RF may promote emotional resilience via enhanced self-efficacy, the lack of causal inference and absence of intervention limit its direct applicability.

Similarly, Weisi and Salari (2024) explored the role of mindfulness as a mediator between reflective practice and professional development in EFL teachers. Although their findings point to the relevance of internal psychological variables, the study’s observational nature and absence of structured intervention reduce its empirical strength.

By contrast, Stacks et al. (2013) adopted a classroom-based approach, analyzing how teachers’ RF influenced behaviors that promote children’s socio-emotional growth. Despite its small sample size and lack of a control group, the study offers practical relevance by situating RF within everyday teaching interactions.

Mayer et al. (2020) contributed to measurement precision by using the Reflective Functioning Scale in structured interviews. The sample size (12 teachers) was small, yet the methodological innovation strengthens the case for integrating diagnostic tools in RF-based teacher interventions.

Among the most methodologically robust studies, Safiye et al. (2023) demonstrated that hypomentalization was linked to emotional exhaustion, whereas hypermentalization predicted reduced burnout and depersonalization in a large Serbian teacher sample. The use of validated RF questionnaires (RFQ) and a strong sample size enhanced external validity, supporting RF as a protective psychological construct.

Schwarzer et al. (2023) further identified that mentalization difficulties—particularly in teachers with insecure attachment styles—contributed to higher stress and burnout vulnerability. Their call for mentalization-based supervision in teacher education provides promising, though still understudied, practical implications.

In sum, while research on RF remains largely correlational or exploratory, the emerging evidence suggests that reflective capacity is trainable and may serve as a key target in psychological interventions aimed at reducing teacher stress and enhancing emotional regulation. Future studies should adopt longitudinal and experimental designs to validate these initial findings and evaluate the impact of RF-focused programs in diverse educational settings.

### The impact of psychological interventions on teachers’ burnout and psychological well-being

Burnout and psychological distress are pervasive issues among teachers, particularly in high-demand environments. The studies reviewed in Supplementary Table 3 consistently indicate that psychological interventions targeting emotional intelligence (EI), mindfulness, and stress regulation strategies can mitigate burnout and enhance overall well-being. Although many studies employed correlational designs, they collectively underscore the protective role of psychological competencies in emotionally demanding school settings.

Lucas-Mangas et al. (2022), using a correlational design with 386 Spanish teachers, found that psychological well-being was a significant predictor of school adaptation. While not intervention-based, the study suggests that fostering EI-related skills may indirectly reduce burnout through improved relational engagement.

Agyapong et al. (2023) provided a narrative review of global psychological interventions, highlighting the effectiveness of mindfulness-based approaches, Cognitive Behavioral Therapy (CBT), and Rational Emotive Behavioral Therapy (REBT). However, the absence of detailed methodological criteria and contextual specificity limits the direct applicability of the findings to specific educational systems.

Sánchez-Pujalte et al. (2023), in a sample of 430 secondary school teachers during the COVID-19 pandemic, identified depressive symptoms as closely linked to emotional exhaustion. This study reinforces the importance of context-sensitive interventions during public health crises and aligns with calls for crisis-responsive mental health programming.

Similarly, Mérida-López and Extremera (2022) found that EI buffered the relationship between perceived stress and intention to leave the profession, especially in the context of student aggression. Though correlational, the study offers a compelling rationale for EI-focused interventions aimed at teacher retention and emotional resilience.

Gao et al. (2013), working with 212 Chinese teachers, demonstrated that EI moderates the effects of work–family conflict on job satisfaction. This finding emphasizes the importance of addressing teachers’ broader psychosocial contexts in burnout prevention strategies.

Wang et al. (2022) adopted a multifactorial model to assess how mindfulness, EI, and coping strategies interact to influence burnout in early childhood educators. Their results showed that adaptive coping and mindfulness mediated the effects of EI on emotional exhaustion—supporting integrated, multi-component interventions over single-strategy approaches.

Johnson and Naidoo (2017) qualitatively evaluated a transpersonal psychology-based program for HIV/AIDS coordinators in high-risk South African schools. The intervention reduced burnout and stress, and while the study’s limited sample constrains generalizability, the rich qualitative data provide insight into the emotional complexity of teacher distress.

Overall, the reviewed studies highlight the relevance of psychological interventions in addressing teacher burnout and promoting well-being, even if limited by methodological variability. Future research should prioritize randomized controlled trials with longitudinal follow-up, ensuring greater internal validity and assessing program feasibility and scalability across diverse educational systems.

### The role of personality traits in the outcomes of psychological interventions for teachers

Personality traits—particularly neuroticism, conscientiousness, and extraversion—have emerged as key moderators of psychological functioning and intervention efficacy in teachers. As summarized in Supplementary Table 4, these traits influence both the outcomes and subjective effectiveness of psychological programs, pointing to the need for individualized and context-sensitive approaches in educational mental health.

Fabbro et al. (2020), in a quasi-experimental study with Italian teachers, found that participants high in conscientiousness and low in neuroticism experienced greater reductions in stress and burnout following a mindfulness-based program. Additionally, the authors reported small but significant shifts in personality traits over time—suggesting that psychological interventions may exert both reactive and transformative effects. This represents a novel contribution, extending the role of interventions beyond immediate coping.

By contrast, Dababneh et al. (2022) used a cross-sectional design to explore how personality dimensions relate to transformational leadership and job satisfaction. They found that extraversion was positively associated with satisfaction, while neuroticism had the opposite effect. Although the study lacked an experimental component, it reinforces the predictive value of personality traits for teacher well-being and leadership readiness.

De Jong et al. (2014) investigated the relationship between personality and disciplinary styles in the classroom. Contrary to expectations, they found no significant association between traits like agreeableness or extraversion and the quality of teacher-student relationships. This suggests that behavioral strategies and classroom context may at times outweigh dispositional traits—highlighting the complexity of personality’s role in teaching effectiveness.

Ding, Zhu, and Yan et al. (2022), in a narrative review of English as a Foreign Language (EFL) teachers, proposed that personality traits interact with communication styles to influence engagement. They suggest that communicative training tailored to specific personality profiles may improve outcomes—a hypothesis yet to be validated experimentally.

The meta-analysis by Kim et al. (2019) confirmed the association between Big Five traits and burnout, showing that emotional stability, conscientiousness, and extraversion are protective factors across cultural contexts. This reinforces the need to incorporate personality screening into teacher support programs and to design interventions that strengthen self-efficacy in line with individual dispositions.

Similarly, Cramer and Binder (2015) identified neuroticism as a consistent predictor of teacher stress in their systematic review. Their findings support the development of targeted interventions to reduce emotional reactivity and enhance emotional regulation—particularly for individuals high in neuroticism.

Overall, the literature underscores the moderating role of personality in shaping both the uptake and impact of psychological interventions. While most studies remain correlational, the convergence of findings calls for future longitudinal and controlled trials that incorporate personality profiling into the design and evaluation of teacher-focused mental health programs.

### The impact of psychological interventions on teachers’ quality of life (QoL)

The studies summarized in Supplementary Table 5 underscore the multifaceted contributions of psychological interventions to enhancing teachers’ Quality of Life (QoL), through mechanisms such as emotional regulation, mindfulness, psychological empowerment, and workload mitigation. While the strength of evidence varies across designs, a narrative appraisal of methodological robustness (Supplementary Table 5) reveals that studies with higher internal validity—such as controlled interventions—offer more actionable recommendations for improving teacher well-being.

Salavera and Urbón (2024), using a correlational design with 344 Spanish pre-service teachers, found that emotional self-regulation was a strong predictor of happiness and subjective well-being. While the study adds conceptual clarity to the relationship between emotional intelligence and QoL, its theoretical contribution is limited by the absence of a direct intervention or experimental controls.

Rahimi et al. (2024), examining 321 Iranian elementary teachers, demonstrated that psychological empowerment mediates the relationship between Quality of Work Life (QWL) and innovative teaching behaviors. Despite its non-interventional nature, the study achieved moderate methodological robustness and offers valuable insights into how well-being connects to organizational transformation.

Da Silva et al. (2024) explored how physical activity buffers the negative effects of workload on QoL among 243 Brazilian teachers. The study, rated moderate-to-high in methodological clarity, found that physical activity was associated with better vitality, social functioning, and mental health—highlighting a practical and scalable intervention strategy.

A more integrative approach was adopted by Corthorn et al. (2024), who investigated the effects of mindfulness on burnout, depression, anxiety, and QoL among Chilean teachers. With robust design features and a broad set of outcome variables, the study supports mindfulness as a comprehensive psychological intervention with multiple downstream benefits.

Di et al. (2023), in a large-scale correlational study involving 936 Chinese kindergarten teachers, emphasized the impact of systemic factors—such as working conditions and access to professional development—on QoL. Though methodologically less rigorous due to its cross-sectional design, the large sample size enhances generalizability and supports the need for policy-level action.

Similarly, Santos et al. (2020) analyzed how somatic symptoms—including voice and musculoskeletal disorders—undermine QoL across all domains. The study, despite its cross-sectional design, was methodologically sound and expanded the definition of teacher well-being to include physical health indicators, supporting the integration of occupational health policies in teacher support frameworks.

A comparative analysis of these findings reveals that the most promising outcomes emerge from studies with higher methodological rigor and clearly defined psychological targets. While individual-level competencies—such as mindfulness and self-regulation—are effective, systemic conditions (e.g., workload, physical health) remain critical determinants. The optimal approach may thus involve a dual strategy: psychological interventions tailored to emotional competencies, alongside institutional reforms addressing the work environment.

Future research should focus on integrated models that combine emotion-focused strategies with workload regulation and occupational health promotion. To strengthen the evidence base, well-powered experimental designs with follow-up assessments are essential, ensuring greater internal validity and supporting the long-term scalability of QoL interventions in diverse educational contexts.

### Limitations of the review

This review presents several limitations that should be carefully considered when interpreting the findings. First, the predominance of cross-sectional and non-randomized designs among the included studies limits the ability to draw causal inferences and to assess the long-term impact of psychological interventions. The scarcity of longitudinal and follow-up studies constrains the review’s capacity to evaluate the durability and sustainability of outcomes over time—factors that are essential for informing educational policy and guiding large-scale implementation.

Second, methodological heterogeneity poses a significant challenge. The studies employed a broad array of instruments to assess emotional intelligence, burnout, reflective functioning, and related psychological constructs. While this reflects the richness of the applied psychological literature, the use of non-standardized or culturally unvalidated tools in some cases compromises comparability, reproducibility, and external validity. This variability was addressed, in part, through the narrative quality appraisal presented in the Supplementary Table 5, which highlighted differences in methodological rigor across studies.

Third, the review reflects a degree of cultural and geographical imbalance. Although the included studies represent diverse regions, a disproportionate number were conducted in Western or high-income contexts, with relatively limited representation from under-resourced or non-Western educational systems. This skew may restrict the generalizability of findings to contexts where cultural, institutional, and pedagogical variables differ markedly.

Fourth, the potential for publication bias must be acknowledged. Peer-reviewed studies reporting statistically significant or favorable results are more likely to be published and indexed in academic databases, which may inflate the apparent effectiveness of interventions. Although this review intentionally excluded gray literature to ensure methodological transparency, it identified relatively few null or negative findings. Future research should encourage the publication of non-significant or adverse results and consider incorporating a broader spectrum of evidence to reduce outcome bias.

Finally, although no formal risk of bias tool (e.g., Cochrane RoB or GRADE) was applied, a structured narrative synthesis was conducted, with results thematically organized across intervention types and outcome domains. The addition of a narrative appraisal of methodological robustness offers an intermediate level of critical evaluation. Nonetheless, future systematic reviews may benefit from integrating formal appraisal tools or mixed-methods coding frameworks to strengthen the assessment of study quality, especially when generating evidence-based recommendations for educational policy and practice.

## Conclusion

This systematic review synthesized evidence from 42 empirical studies exploring the impact of psychological interventions on teachers’ emotional intelligence, reflective functioning, burnout, and quality of life. The results suggest that interventions such as mindfulness training, emotional skills development, and psychological empowerment can contribute meaningfully to enhancing teachers’ emotional well-being, reducing burnout, and improving professional resilience and adaptability.

A central implication of these findings is the importance of tailoring psychological interventions to individual characteristics. Personality traits—particularly neuroticism, conscientiousness, and emotional stability—were found to influence the effectiveness of such programs (Kim et al., 2019; Fabbro et al., 2020). This reinforces the need to move beyond one-size-fits-all models and to adopt more differentiated approaches that consider individual variability in emotional functioning and responsiveness to interventions.

In parallel, the review highlights the critical role of organizational and contextual factors. Studies consistently demonstrated that working conditions, perceived institutional support, and physical health considerations directly affect teachers’ psychological functioning (Di et al., 2023; Santos et al., 2020). Therefore, effective interventions should combine individual-level psychological resources with systemic strategies aimed at improving workload regulation, school climate, and occupational health support.

Another relevant finding concerns the ripple effects of psychological interventions. Programs that foster emotional intelligence and reflective functioning can benefit not only teachers, but also students and the wider educational environment, by improving interpersonal dynamics and classroom atmosphere. For example, mindfulness-based approaches have been associated with reduced stress and more positive learning climates (Corthorn et al., 2024), suggesting that promoting teacher well-being may yield broader gains for educational quality and school culture.

The review also underscores the importance of preparedness and resilience in times of crisis. The COVID-19 pandemic significantly exacerbated emotional exhaustion among teachers, underscoring the need for interventions that are both flexible and evidence-based, and that equip educators to cope with both daily challenges and exceptional disruptions (Sánchez-Pujalte et al., 2023).

Taken together, the findings support the need for a holistic, multilevel framework for promoting teacher well-being—one that integrates psychological resources with institutional reforms and policy development. Future research should prioritize robust, longitudinal, and implementation-focused designs capable of evaluating long-term efficacy, cultural adaptability, and scalability. Aligning individual-level support with systemic transformation will be essential for sustaining teacher flourishing and improving educational outcomes.

### Implications for research and practice

This review highlights the need to align psychological interventions with both individual teacher profiles and systemic educational reforms. Programs should be designed with attention to personality traits, emotional competencies, and contextual stressors. Policymakers and school administrators are encouraged to promote scalable, evidence-based models that support teacher well-being through integrated strategies. Future studies should further investigate how such interventions can be sustainably implemented across diverse educational settings.

## Data Availability

The original contributions presented in the study are included in the article/Supplementary material, further inquiries can be directed to the corresponding author.

## Glossary

ARR: Assessment of Representational Risk
CBT: Cognitive Behavioral Therapy
ECR-RD8: Expectations in Close Relationships-Revised Questionnaire
EI: Emotional Intelligence
FFMQ: Five Facet Mindfulness Questionnaire
IBSR: Inquiry-Based Stress Reduction
INTE: Emotional Intelligence Questionnaire (Jaworowska & Matczak)
MBI: Maslach Burnout Inventory
MBI-ES: Maslach Burnout Inventory-Educators Survey
MM: Mindfulness Meditation
PDI: Parent Development Interview
PDI-R/T: Parent Development Interview-Revised for Teachers
PHQ-9: Patient Health Questionnaire
QOL: Quality of Life
QWLSKT: Quality of Work Life Scale for Kindergarten Teachers
REBT: Rational Emotive Behavior Therapy
RF: Reflective Functioning
RFQ: Reflective Functioning Questionnaire
SCQ: Social Competence Questionnaire
SEL: Social and Emotional Learning
SF-36: Short Form Health Survey 36
SMART: Stress Management and Resilience Training
TICS: Trier Inventory of Chronic Stress
TMMS-24: Trait Meta-Mood Scale-24 items
TSI: Teacher Stress Inventory
WHOQOL-BREF: World Health Organization Quality of Life-Brief version
WFC: Work-Family Conflict
QWL: Quality of Work Life
